# Objectively Measured Photoaging and Skin Cancer in Organ Transplant Recipients

**DOI:** 10.2340/actadv.v106.adv-2026-0417

**Published:** 2026-09-30

**Authors:** Stine R. Wiegell, Peter A. Philipsen, Susanne K. Kjær, Jay F. Nash, Peter Bjerring, Merete Hædersdal

**Affiliations:** 1 Department of Dermatology, Copenhagen University Hospital Bispebjerg, Copenhagen, Denmark; 2 Department of Clinical Medicine, Faculty of Health and Medical Science, University of Copenhagen, Copenhagen, Denmark; 3 Unit of Virus, Lifestyle and Genes, Danish Cancer Institute, Copenhagen, Denmark; 4 Juliane Marie Centre, Rigshospitalet, University of Copenhagen, Copenhagen, Denmark; 5 Global Product Stewardship, The Procter & Gamble Company, Mason, Ohio, United States; 6 Department of Dermatology, Aalborg University Hospital, Aalborg, Denmark

**Keywords:** Solid organ transplant recipients, photoaging, photoaging phenotype, photodamage, actinic keratosis, keratinocyte skin cancer

## Abstract

Solid organ transplant recipients (SOTRs) have an increased risk of keratinocyte skin cancer (KC) due to lifelong immunosuppression. Photoaging, reflecting cumulative ultraviolet (UV) exposure, may serve as a biomarker of KC risk. This study characterized objective photoaging features in SOTRs and their association with age, photodamage, photoaging phenotype, actinic keratoses (AKs) and KC history. In 251 SOTRs, VISIA imaging showed significant age-related increases in photoaging features; median red feature scores reflecting erythema and telangiectasia were 44 AU (arbitrary units) in patients >60 vs 22 AU in those <60. Higher clinical photodamage severity corresponded to higher VISIA scores, especially red features and visible spots. The atrophic photoaging phenotype, observed in 60% of patients, showed higher red features (40 AU) than the hypertrophic type (27 AU). Patients with AKs had elevated UV spots and red features. A history of KC (32% of participants) was associated with older age, longer time since transplantation, fairer skin type, more severe photodamage, higher VISIA scores, especially red features (45 AU vs 29 AU) and greater pigmentation on sun-exposed sites. Objective imaging identified photoaging features associated with cumulative UV damage. Further studies should evaluate their predictive value for KC development to improve individualized risk stratification and surveillance strategies in this high-risk population.

**SIGNIFICANCE**

Solid organ transplant recipients have a markedly increased risk of skin cancer due to lifelong immuno-suppression. Our study demonstrates that objective skin imaging can identify specific features of photoaging that are associated with actinic keratoses and a history of keratinocyte skin cancer. Quantitative assessment of photoaging, especially of vascular changes, offers valuable information that may complement current tools for assessing individual risk beyond traditional clinical evaluation. Further studies are needed to determine whether these measures can predict future skin cancers and guide tailored monitoring for organ transplant recipients.

Organ transplantation significantly extends life and improves quality of life for patients with organ failure (1, 2). Advances in medical science have markedly improved survival rates among transplant recipients, primarily through the optimization of immunosuppressive therapy, which prevents rejection of the transplanted organ (3).

However, lifelong immunosuppression substantially increases the risk of skin cancer by impairing the body’s ability to repair UV-induced DNA damage (4). Solid organ transplant recipients (SOTRs) face up to an 11-fold increased risk of squamous cell carcinoma and a 6-fold risk of basal cell carcinoma, with keratinocyte skin cancer mortality 20 times higher than in the general population (5, 6).

Preventive strategies and early detection are essential to improve outcomes, as skin cancer poses a serious threat to this patient group and accounts for 5% of overall mortality among transplant recipients (7). Consequently, routine dermatologic surveillance is strongly recommended for SOTRs (8, 9). Given the growing number of transplant recipients, individualized skin cancer screening based on personalized risk assessment is increasingly important (9, 10). Risk stratification scoring systems such as the SUNTRAC tool incorporate key predictive variables including sex, race, age at transplantation, pre-transplant history of skin cancer and type of immunosuppression (10). The use of SUNTRAC stratification of SOTRs has shown to improve screening rates and early detection of KC with the potential of reducing morbidity and healthcare costs (5).

Photoaging reflects changes in the skin following chronic exposure to solar ultraviolet radiation (11). Among white northern Europeans, facial photoaging can be divided into 2 clinical phenotypes: hypertrophic photoaging (HP) and atrophic photoaging (AP) (11). Deep, coarse wrinkles dominate individuals with HP and those with AP have unwrinkled skin with pronounced telangiectasia. Studies have suggested that individuals with AP are at increased risk of developing actinic keratoses and basal cell carcinomas (11, 12). The association between the photoaging phenotype and skin cancer in organ transplant recipients has not been evaluated.

Since photoaging reflects cumulative UV damage, its quantification could improve individual risk prediction for UV-related skin cancer (13). Traditionally, photoaging severity has been assessed using subjective clinical scoring systems (14). However, objective, non-in-vasive digital imaging techniques now enable more accurate and reproducible evaluation (14). The VISIA Imaging System is a validated platform that employs multispectral imaging and algorithmic analysis to quantify features of photodamage such as wrinkles, UV-spots and vascular changes with high sensitivity (15).

The aim of this study was to describe objective features of photoaging in the high-risk group of organ transplant recipients and describe these features in association with age, photodamage, photoaging phenotype, actinic keratosis and history of keratinocyte skin cancer.

## METHODS

### Study design

This was a single-centre cross-sectional study conducted at Department of Dermatology, Bispebjerg University Hospital in Copenhagen, Denmark. Patients were recruited during their routine full skin examinations, performed at various intervals throughout the year based on their clinical risk and skin cancer history. Patients were included from March 2022 to September 2025. Main inclusion criteria were SOTRs (kidney, liver, lung and heart), stable immunosuppression for at least 3 months and no signs of acute graft rejection.

The study was conducted in accordance with the ethical principles of the Declaration of Helsinki. Signed informed consent was obtained from all participants prior to entry into the study. The study was approved by the local ethics committee (H-21038387) and the Danish Data Protection Agency and registered on clinicaltrials.gov (NCT05284877).

### Study procedure

An in-clinic full skin examination was performed by specialists in dermatology to evaluate the degree of photodamage (minimal, mild, moderate and severe) and the presence of actinic keratoses and keratinocyte skin cancer. Based on standardized clinical photographs, a dermatologist further classified the photoaging phenotype as predominantly atrophic, hypertrophic or no signs of photoaging. The atrophic phenotype was characterized by unwrinkled skin with prominent telangiectasia, whereas the hypertrophic phenotype was defined by deep, coarse wrinkles. History of keratinocyte skin cancer (KC) was obtained from the Danish Pathology Data Bank (Patobank) and included basal cell carcinoma (BCC) and squamous cell carcinoma (SCC).

The VISIA Imaging System (Canfield Scientific Inc., Parsippany, NJ, USA) was used to capture standardized, high-resolution facial images under controlled lighting. VISIA images were taken frontally and at 33° angles from the left and right side. The images were then analysed using the intrinsic VISIA software (version 10.0) for wrinkles, texture, UV spots, brown spots, visible spots and red features (**Fig. 1** and Video S1) (15). Ultraviolet lighting was used to generate a UV spot image highlighting solar lentigines due to the selective absorption of UV light in epidermal melanin (15). Cross-polarized lighting was used to capture the brown spots and red features by measuring the amount of melanin and haemoglobin in the skin. Melanin serves as a proxy for brown discolouration and cross-polarized light can detect deeper deposition of melanin than UV light. Haemoglobin serves as a measure of the amount of redness in the skin including background erythema, telangiectasias and vascular lesions (15). Finally, using standard flash it is possible to detect wrinkles, texture (fine lines), and visible spots (including lentigines, acne scars and vascular lesions) (15). The VISIA software generates an absolute score for each feature reflecting the total size, area and intensity of the skin criterion (15). The mean of the absolute score from the 3 facial images was calculated. For wrinkles, counts rather than scores were used, and the total number of wrinkles from the 3 facial images was summed.

**Fig. 1.**
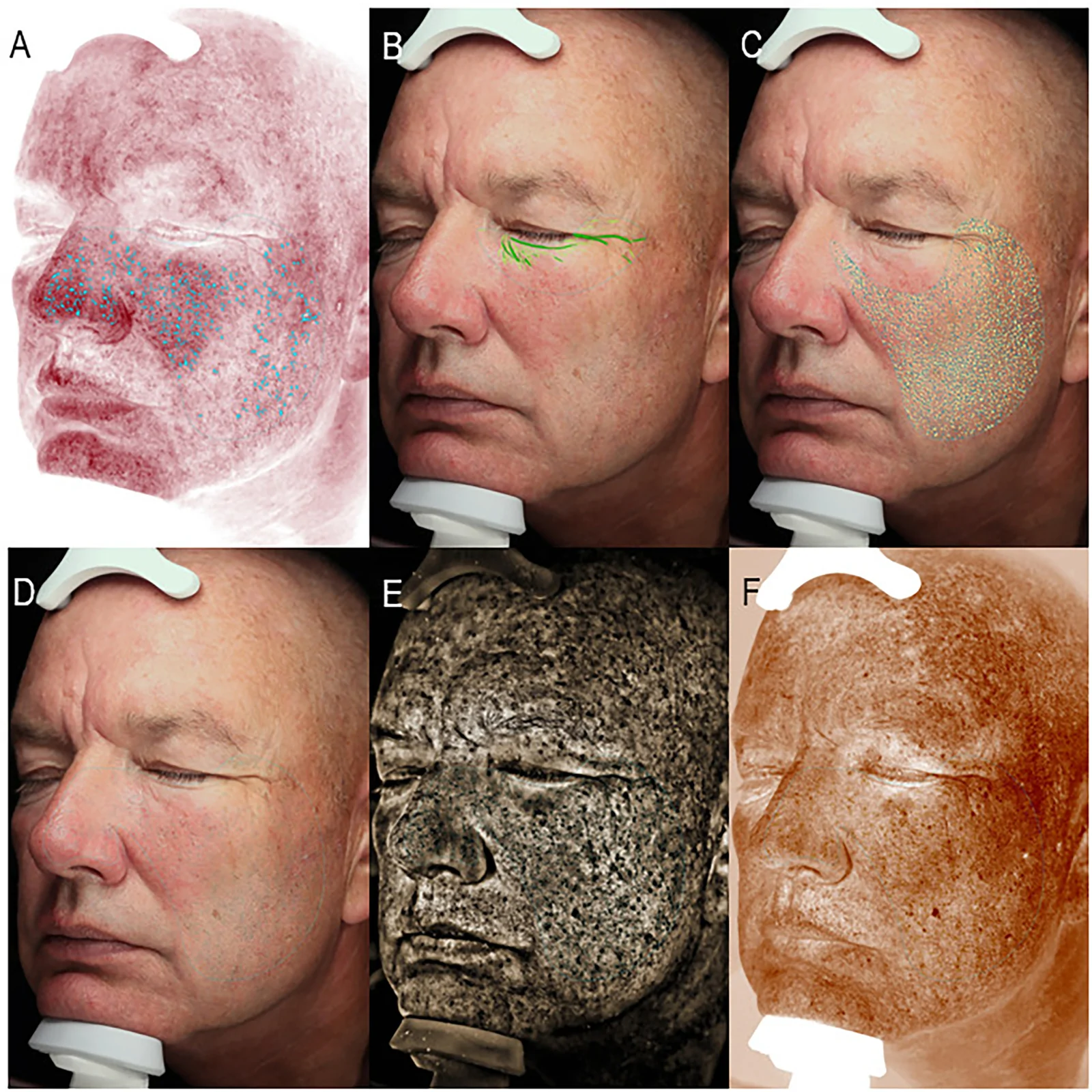
VISIA images of the right facial view showing: (A) red features (cross-polarized lighting), (B): wrinkles (standard flash), (C) texture (standard flash), (D) visible spots (standard flash), (E) UV spots (ultraviolet lighting), (F) brown spots (cross-polarized lighting).

Pigmentation and Individual Typology Angel (ITA) were objectively assessed in 4 standardized locations on right cheek, right inner upper arm, back of the left shoulder and left upper buttock using a skin colourimeter (DSM IV, Cortex Technology, Hadsund, Denmark) (Video S2). The skin colourimeter measures the intensity of light reflected from chromophores in the skin such as haemoglobin and melanin which allows quantification of erythema and pigmentation (16). The DSM colourimeter uses the CIE L* a* b* colour space system and calculates the ITA, an objective classification of skin colour, in which individuals with lighter skin pigmentation have a higher ITA value than darker pigmented individuals. The measurements on the buttocks and inner upper arm served as proxy for constitutive skin colour and measurement on the cheek and shoulder for facultative skin colour.

### Statistics

Descriptive statistics were used to evaluate SOTRs demographics as well as objective measurements of photoaging. All parameters were compared for patients with and without AK, KC, SCC and BCC as well as young (<60 years old) vs old patients (>60 years old) using Mann-Whitney Test or χ^2^ test. All parameters were compared for photoaging phenotype and the 4 categories of clinical photodamage using Kruskal-Wallis test.

We considered 2-tailed *p*-values < 0.05 statistically significant. All analyses were performed using SPSS version 29 (IBM, New York, NY, USA).

## RESULTS

A total of 291 SOTRs were included in the study. Forty patients (14%) were excluded from the analysis of objective photoaging due to the use of facial sunscreen or makeup containing sunscreen during the evaluation, which interfered with VISIA imaging resulting in a final cohort of 251 OTRs. The excluded SOTRs were mainly women (78%), mostly included in late spring or summer, and their median age was approximately 10 years younger than that of the included population (Fig. S1). All VISIA-derived scores were significantly lower among patients wearing facial sunscreen or make-up compared with those who did not. Facial pigmentation was also lower in the sunscreen group, whereas no significant differences were observed in pigmentation measurements on the shoulder between the 2 groups (**Table I**).

**Table I.** Age, pigmentation and photoaging features in patients with and without facial sunscreen or make-up

	Sunscreen *n*=40 Median (IQR)	No sunscreen *n*=251 Median (IQR)	*p*-value
Age	49 (37–58)	59 (49–67)	0.0003
Melanin cheek, AU	33.5 (31.0–35.3)	35.7 (33.7–37.7)	0.0001
Melanin shoulder, AU	34.6 (31.4–36.4)	34.5 (32.7–36.5)	0.55
UV spots score (mean), AU	13.0 (8.8–19.4)	33.4 (28.4–38.5)	<0.0001
Red features score (mean), AU	17.0 (10.5–27.4)	32.0 (20.2–48.6)	<0.0001
Brown spots score (mean), AU	16.6 (12.6–22.6)	24.8 (19.7–30.3)	<0.0001
Visible spots score (mean), AU	28.4 (23.3–37.8)	39.6 (34.0–44.5)	<0.0001
Wrinkles count (sum), AU	271 (216–351)	240 (193–277)	0.012
Texture score (mean), AU	10.0 (5.3–17.6)	15.9 (10.5–22.5)	0.0008

Subjects evaluated while wearing sunscreen were excluded from the final analysis. UV spots, red features, brown spots, visible spots and texture were a mean of the score from 3 facial images. Wrinkles was a sum the count from 3 facial images.

AU: Arbitrary units; IQR: interquartile range.

Our final cohort of 251 SOTRs comprising 92 females and 159 males. The median age of participants was 59 years (interquartile range (IQR): 49–67 years). Median age at transplantation was 49 years (IQR: 36–58 years) (**Table II**). Patients had been on immunosuppressive treatment due to transplantation up to 42 years, although the median duration of immunosuppression was 8 years (IQR: 3–13 years).

**Table II.** Baseline characteristics for all included patients not wearing facial sunscreen or makeup

		Female SOTRs *n*=92	Male SOTRs *n*=159	*p*-value
Age, years median (IQR)		54 (42–62)	61 (52–69)	<0.0001
Years with transplantation, median (IQR)		7 (2-13)	9 (5-14)	0.077
Age at transplantation, years median (IQR)		46 (30–56)	51 (40–59)	0.004
Transplant organ no. patients (percentage)	Kidney Liver Lung Hearth Multiple	45 (49%) 26 (28%) 11 (12%) 9 (10%) 1 (1%)	104 (65%) 26 (16%) 13 (8%) 13 (8%) 3 (2%)	0.051
Fitzpatrick skin type no. patients (percentage)	I II III IV	7 (8%) 47 (51%) 33 (36%) 5 (5%)	2 (1%) 91 (57%) 63 (40%) 3 (2%)	0.021
Clinical severity of photodamage no. patients (percentage)	Minimal Mild Moderate Severe	55 (60%) 25 (27%) 10 (11%) 2 (2%)	64 (40%) 40 (25%) 51 (32%) 4 (3%)	0.001
Photoaging phenotype no. patients (percentage)	No Atrophic Hypertrophic	16 (17%) 39 (42%) 37 (40%)	10 (6%) 112 (70%) 37 (23%)	<0.0001
Clinical actinic keratosis no. patients (percentage)		19 (21%)	66 (42%)	0.0008
Keratinocyte skin cancer no. patients (percentage)	No BCC SCC BCC+SCC	73 (79%) 12 (13%) 0 (0%) 7 (8%)	97 (61%) 31 (19%) 8 (5%) 23 (14%)	0.009

AU: Arbitrary units; BCC: Basal cell carcinoma; IQR: interquartile range; SCC: squamous cell carcinoma; SOTRs: Solid organ transplant recipients.

Objective measurements of facial photoaging using VISIA Skin Analysis were dependent on age (**Fig. 2** and **Table III**). Dividing the cohort into patients less than 60 years and patients 60 years and older showed an increase in all photoaging features except wrinkle count in the older population compared to the younger population (Table III). Differences between the older and younger population were largest for UV spots with a median score of 35.9 (IQR: 31.0–39.9) compared to 31.7 (IQR: 26.8–35.8) and red features with a median score of 43.9 (IQR: 32.4–54.0) compared to 22.4 (IQR: 15.1–34.4). Wrinkle counts were significantly higher in the younger population with a median count of 246 (IQR: 196–290) compared to 226 (IQR: 191–262) in the older population (*p=*0.01). The older population were more often men (*p=*0.001), were older at transplantation (*p<*0.0001), more often had fair Fitzpatrick skin type (*p=*0.003), a more severe degree of clinical photodamage (*p<*0.0001) and more often actinic keratoses (*p<*0.0001).

**Fig. 2.**
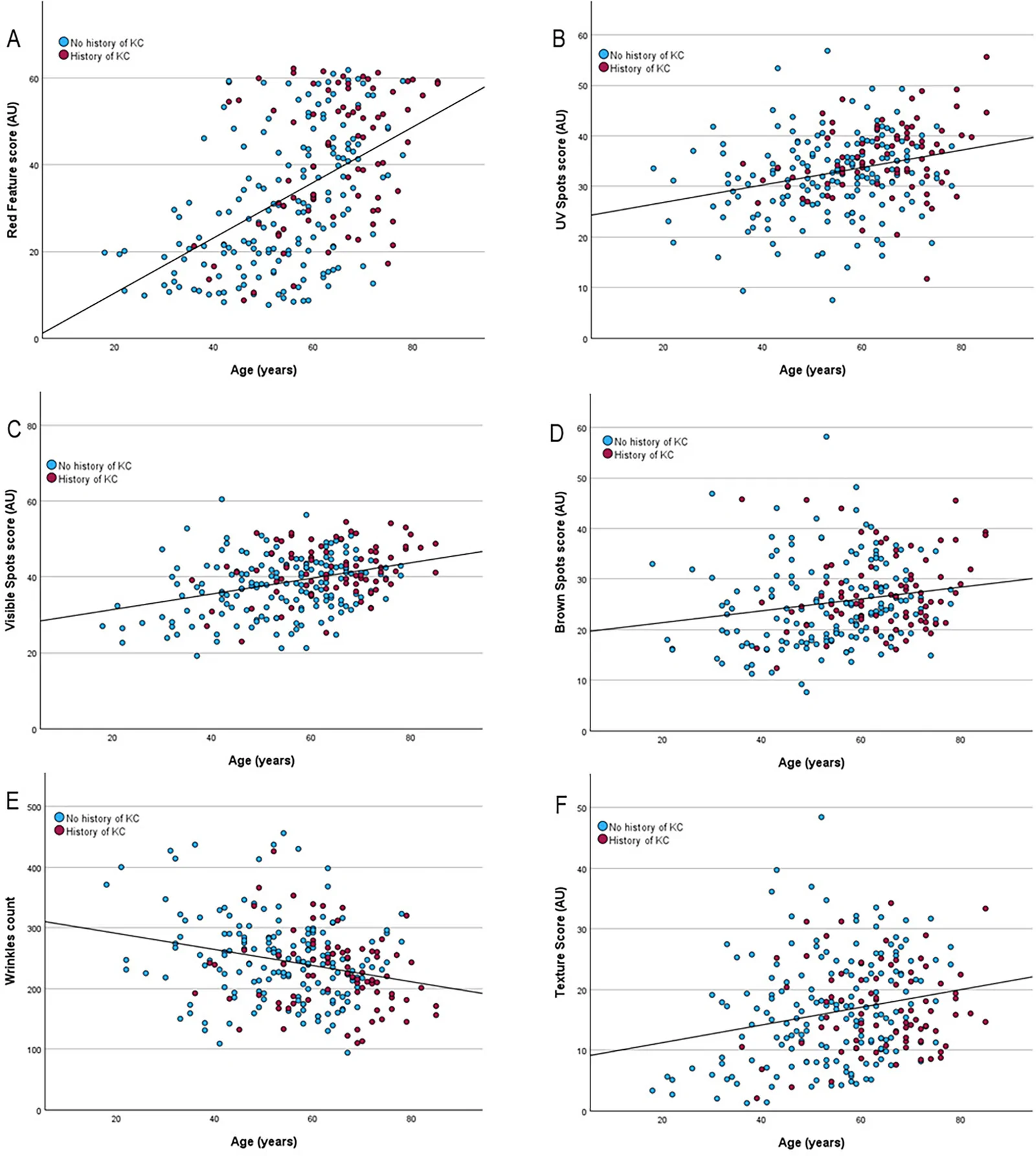
Objective photoaging features, based on VISIA Skin Analysis, increased with age except wrinkles count which decreased with age. Red dots representing patients with a history of keratinocyte skin cancer and blue dots patients with no history of keratinocyte skin cancer.

**Table III.** Pigmentation and objective photoaging in patients younger than 60 years compared to patients 60 years or older

	<60 years median (IQR) *n*=132	≥60 years median (IQR) *n*=119	*p*-value
Ratio melanin shoulder/buttocks, AU	1.1 (1.0–1.2)	1.2 (1.1–1.3)	<0.0001
Diff ITA shoulder/buttocks, AU	2.5 (-7.4–13.7)	8.2 (1.4–19.9)	0.001
UV spots score (mean), AU	31.7 (26.8–35.8)	35.9 (31.0–39.9)	<0.0001
Red features score (mean), AU	22.4 (15.1–34.4)	43.9 (32.4–54.0)	<0.0001
Brown spots score (mean), AU	23.2 (17.8–29.0)	26.3 (22.5–31.9)	0.0004
Visible spots score (mean), AU	37.9 (30.4–42.7)	41.2 (36.2–45.2)	<0.0001
Wrinkles count (sum), AU	246 (196–290)	226 (191–262)	0.01
Texture score (mean), AU	15.1 (8.0–21.0)	16.5 (11.9–23.0)	0.01

AU: Arbitrary units; IQR: interquartile range.

The clinical assessed degree of photodamage as summarized in Table II, was categorized as minimal in 119 patients (47%), mild in 65 patients (26%), moderate in 61 patients (24%) and severe in 6 patients (2%). Male SOTRs had higher photodamage than female SOTRs (*p*=0.001). Objective photoaging features increased with increasing severity of clinical photodamages especially for red features (*p<*0.0001) and visible spots (*p<*0.0001). Patients with minimal clinical photodamage had a median red features score of 22.4 (IQR: 14.8–35.1) compared to 55.9 (IQR: 45.8–60.8) in patients with severe clinical photodamage. The same was seen for visible spots with a median visible spot score of 36.9 (IQR: 29.5–42.3) in patients with minimal clinical photodamage and 48.3 (IQR: 43.8–51.7) in patients with severe clinical photodamage (**Fig. 3**).

**Fig. 3.**
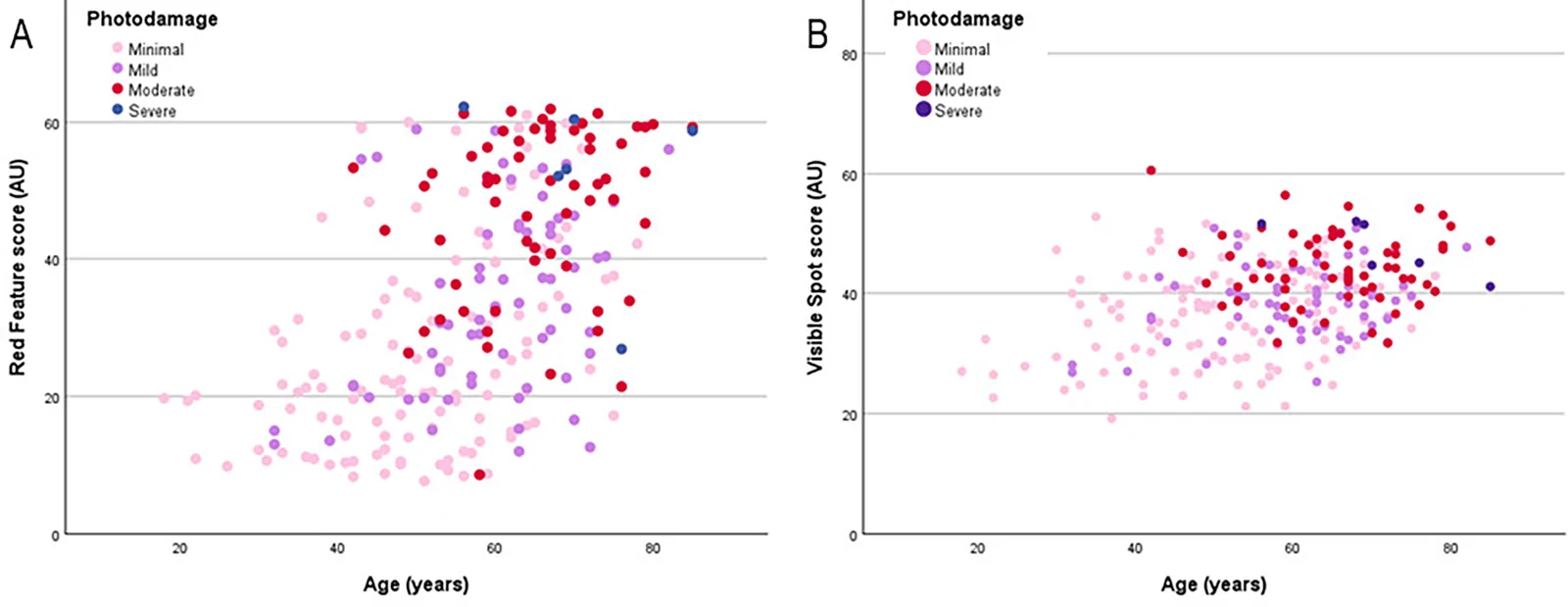
Clinical photodamage associated with red features (A) and visible spots (B), based on VISIA Skin Analysis, and age (A).

The atrophic phenotype of photoaging was seen in 151 patients (60%) and 74 patients (30%) had the hypertrophic type (Table II). The remaining 26 patients had no photoaging (10%). Patients with no photoaging were significantly younger (median age 38 years) than patients with either atrophic (median age 59 years) or hypertrophic photoaging (median age 63 years) (*p<*0.0001). Male patients (70%) more often had atrophic photoaging than female patients (42%). Patients with atrophic photoaging had a median red features score of 39.8 (IQR: 27.5–52.5) compared to 26.7 (IQR: 15.2–43.9) in patients with hypertrophic photoaging (*p<*0.0001) (**Fig. 4**).

**Fig. 4.**
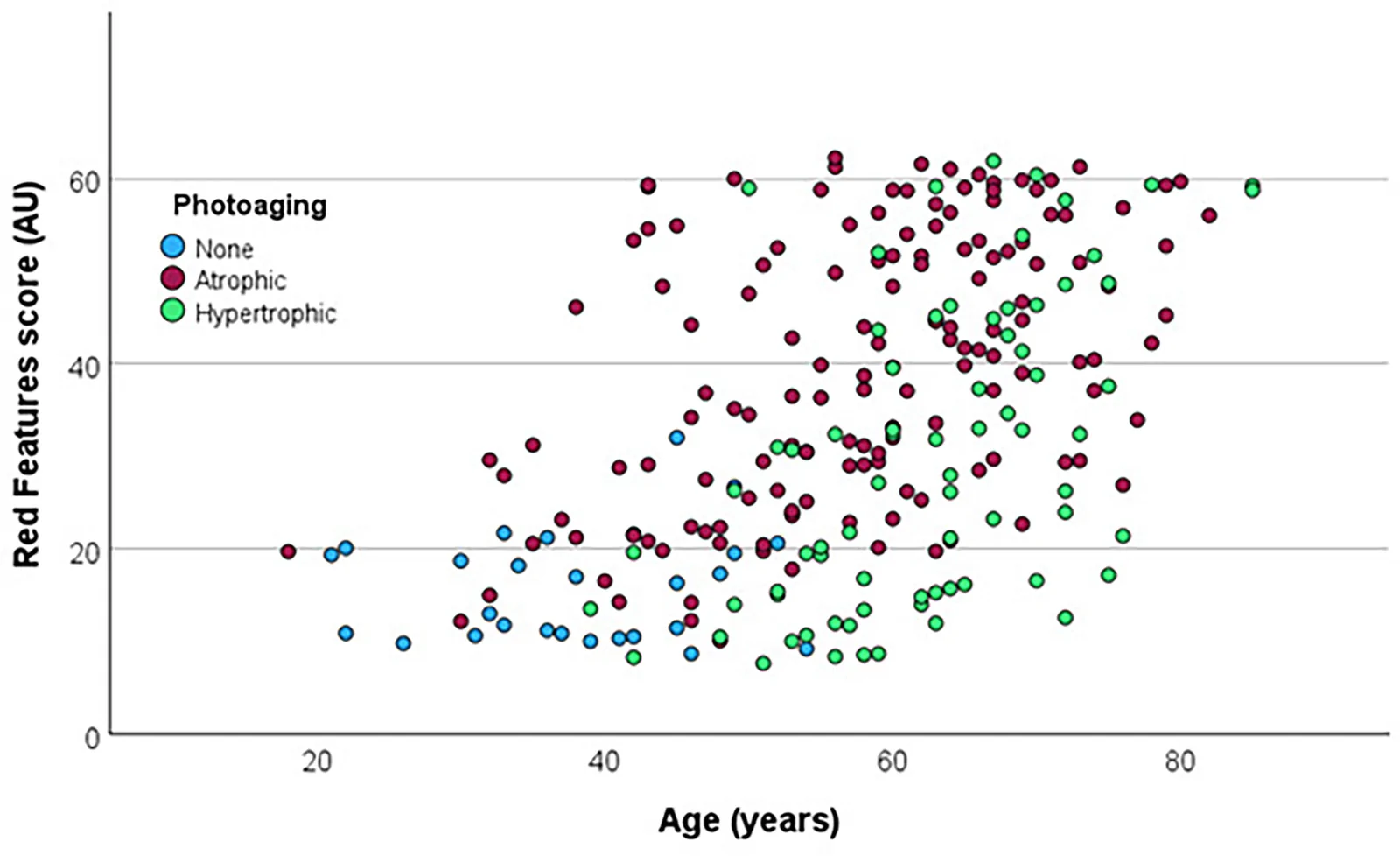
Photoaging phenotype associated with age and red features score based on VISIA Skin Analysis. Atrophic phenotype characterized by unwrinkled skin with prominent telangiectasia and hypertrophic phenotype by deep, coarse wrinkles. Blue dots: no photoaging. Red dots: primarily atrophic photoaging. Green dots: primarily hypertrophic photoaging.

Clinical skin examination revealed actinic keratoses in 85 patients (34%). Patients with actinic keratoses were older (*p<*0.0001), transplanted for a longer time (*p<*0.0001), more often male (*p=*0.001), more fair Fitzpatrick skin type (*p=*0.002) and with more clinical photodamaged (*p<*0.0001) than patients without actinic keratoses. Objective measurements of photoaging showed significantly higher UV spot scores (*p<*0.0001), red feature scores (*p<*0.0001), visible spots (*p=*0.001) and brown spots (0.03) in patients with actinic keratosis compared to patients without.

In total, 81 patients (32%) had a history of KC. Thirty-eight patients (15%) were diagnosed with SCC, and 73 patients (29%) with BCC and 30 of these patients had a history of both BCC and SCC (12%). Seventeen patients (7%) had developed skin cancer before transplantation and 9 of these patients (53%) developed additional carcinomas after transplantation.

Patients with a history of KC were older (median age 66 vs 54 years, p<0.0001) and more often men (76% vs 57%, p=0.009) compared to patients without KC. In addition, patients with skin cancer had on average been transplanted for 5 more years (*p<*0.0001) and were 6 years older at transplantation (*p=*0.02) compared to patients without skin cancer. Patients with a history of skin cancer had more severely photodamaged skin (*p<*0.0001), more fair Fitzpatrick skin type (*p=*0.01) and more often actinic keratoses at clinical evaluation (*p<*0.0001) compared to patients without skin cancer.

When comparing SOTRs with a history of KC to those without, the KC group exhibited a higher pigmentation/ITA ratio on the shoulder relative to the buttocks. They also had a significantly higher VISIA score for UV spots, red features, brown spots and visible spots and a lower wrinkle count (**Table IV**).

**Table IV.** Age and photoaging features in patients without a history of keratinocyte skin cancer compared to patients with a history of keratinocyte skin cancer, squamous cell carcinoma or basal cell carcinoma

	No KC Median (IQR) *n*=170	KC Median (IQR) *n*=81	*p*-value	SCC Median (IQR) *n*=38	*p*-value	BCC Median (IQR) *n*=73	*p*-value
Age, years	56 (46–63)	66 (56–72)	<0.0001	69.5 (63-74)	<0.0001	65 (56–73)	<0.0001
Pigmentation shoulder, AU	34.3 (32.3–36.3)	34.9 (33.2–37.2)	0.078	35.4 (33.7–37.6)	0.017	34.9 (33.3–37.2)	0.060
Ratio pigmentation shoulder/Buttocks, AU	1.11 (1.02–1.20)	1.17 (1.08–1.25)	0.005	1.18 (1.09–1.28)	0.003	1.17 (1.08–1.25)	0.0062
Difference ITA shoulder/Buttocks, AU	3.5 (-5.5–14.2)	7.7 (-0.4–18.2)	0.017	21.5 (-0.1–24.4)	0.026	8.1 (-0.4–18.1)	0.018
UV spots, Score (mean), AU	32.4 (27-38)	35.4 (32-40)	0.0005	39.0 (33-42)	<0.0001	34.8 (31-40)	0.0012
Red features, Score (mean), AU	28.9 (18-44)	44.6 (29-55)	<0.0001	51.9 (44-59)	<0.0001	40.2 (27-54)	<0.0001
Brown spots, Score (mean), AU	24.1 (19-30)	25.7 (22-31)	0.009	27.6 (23-31)	0.006	26.4 (22-32)	0.0072
Visible spots, Score (mean), AU	38.1 (32-43)	41.7 (38-48)	<0.0001	43.0 (39-48)	<0.0001	41.7 (38-48)	<0.0001
Wrinkles, Count (mean), number	243 (201–286)	225 (184–261)	0.034	211 (166–253)	0.0046	226 (186–261)	0.058

AU: arbitrary units; BCC: basal cell carcinoma; IQR: interquartile range; KC: Keratinocyte skin cancer; SCC: squamous cell carcinoma.

Similar significant differences were observed in subgroup analyses of patients with and without history of SCC and BCC, with the exception that wrinkle count did not differ significantly between patients with and without BCC (Table IV). In patients with a history of SCC especially red features were increased compared to patients without KC with a median score of 51.9 (IQR: 12–62 compared to 28.9 (IQR: 8–62) (*p<*0.0001).

## DISCUSSION

In this single-centre cross-sectional study of SOTRs, we objectively characterized facial photoaging using VISIA Imaging System. Our findings demonstrate that objective photoaging features are associated with established risk factors for keratinocyte skin cancer in SOTRs and suggest that quantitative assessment of photoaging may add value to individualized skin cancer risk stratification in this high-risk population.

Consistent with prior studies in immunocompetent populations, VISIA measures of photoaging increased with chronological age, particularly UV spots and red features, reflecting cumulative ultraviolet exposure and progressive vascular damage (Fig. 2) (17, 18). Notably, wrinkle counts were paradoxically higher in younger SOTRs, a finding that does not align with a study in immunocompetent populations where wrinkle count increased up to the age of 60 years and then remained stable until the age of 89 years (18). Older SOTRs were more likely to exhibit the atrophic photoaging phenotype, which is characterized by relative absence of deep wrinkles but prominent telangiectasia and erythema, features that were captured by elevated red features scores on VISIA imaging. This underscores the limitation of relying solely on wrinkle-based assessments of photoaging and highlights the importance of multidimensional, objective evaluation.

A key observation of this study is the predominance of the atrophic photoaging phenotype, which was present in 60% of recipients and was associated with significantly higher red feature scores compared to hypertrophic photoaging. Prior studies in immunocompetent populations have suggested that atrophic photoaging is associated with increased risk of actinic keratoses and basal cell carcinoma (11, 12). Our findings extend these observations to organ transplant recipients, a population in whom this association had not previously been evaluated.

The degree of clinically assessed photodamage correlated strongly with objective VISIA-derived parameters, particularly red features and visible spots (Fig. 3). This concordance supports the construct validity of digital imaging as a reproducible and sensitive method for quantifying cumulative UV damage. Importantly, the steep increase in red feature scores across increasing photodamage categories suggests that vascular changes may be among the most sensitive indicators of severe photodamage in SOTRs. Given that erythema and telangiectasia are hallmarks of atrophic photoaging, these findings further link vascular photoaging to skin cancer susceptibility.

Actinic keratoses were present in one-third of the population and were associated with older age, longer transplant duration, fair skin type and more severe clinical photodamage, in line with existing literature (19). Importantly, patients with actinic keratoses exhibited significantly higher VISIA scores for UV spots, red features, brown spots and visible spots. These findings suggest that objective photoaging features capture subclinical photodamage relevant to field cancerization and as actinic keratoses are strongly associated with the development of KC, measurements of photoaging may help identify patients at increased risk of skin cancer (19, 20).

A history of keratinocyte skin cancer was present in nearly one-third of participants, emphasizing the substantial burden of skin cancer in this high-risk population of SOTRs. Patients with a history of KC displayed significantly higher objective photoaging scores across multiple parameters, particularly UV spots and red features, and a lower wrinkle count, further supporting an association with the atrophic photoaging phenotype. The observation that red feature scores were especially elevated in patients with a history of SCC is noteworthy, given the markedly increased risk of SCC in SOTRs compared with immunocompetent individuals (5). These findings suggest that vascular photoaging may be a particularly relevant marker of SCC risk in immunosuppressed patients.

Objective pigmentation measurements further demonstrated that patients with a history of KC had a higher facultative-to-constitutive pigmentation ratio, indicating greater UV-induced pigmentation relative to baseline skin colour. This shows tanning in fair skinned patients may contribute to skin cancer susceptibility and could be integrated into future risk prediction models.

### Strengths and limitations

This study has several strengths, including the relatively large, well-characterized cohort, standardized dermatologic examinations, linkage to national pathology data, and the use of validated objective imaging and colourimetric techniques. However, limitations should be acknowledged. The single-centre design may limit generalizability, however the objective measurements of photodamage are reproducible and not dependent on the investigator making the results applicable to other centres. The exclusion of patients wearing sunscreen or cosmetic products may have introduced selection bias, as a younger and predominantly female subgroup was excluded. Additionally, the study was conducted in a predominantly fair-skinned Northern European population, which may limit generalizability to more diverse ethnic groups. Finally, the cross-sectional nature of the measurements limits our ability to establish causal relationships between photodamage features and skin cancer development. Future studies should investigate the predictive value of these imaging features in longitudinal cohorts.

### Conclusion

Objective measures of photoaging are strongly associated with actinic keratoses and history of keratinocyte skin cancer in SOTRs and reflect clinically meaningful differences in photoaging phenotype. Quantitative assessment of photoaging, particularly vascular features captured by red feature scores, may provide valuable complementary information to existing clinical risk stratification tools. Longitudinal studies are warranted to determine whether objective photoaging metrics can predict future skin cancer development and to evaluate their potential role in personalized surveillance strategies for transplant recipients.

## Data Availability

The data that support the findings of this study are available from the corresponding author upon reasonable request.
