# Supplementary material for: Objectively Measured Photoaging and Skin Cancer in Organ Transplant Recipients
Source: Acta Derm Venereol. 2026 Sep 30;106:0417. doi: 10.2340/actadv.v106.adv-2026-0417 (PMC13625172; doi:10.2340/actadv.v106.adv-2026-0417)
Supplement: Supplementary file 1 [file ActaDv-106-0417-s0001.pdf]

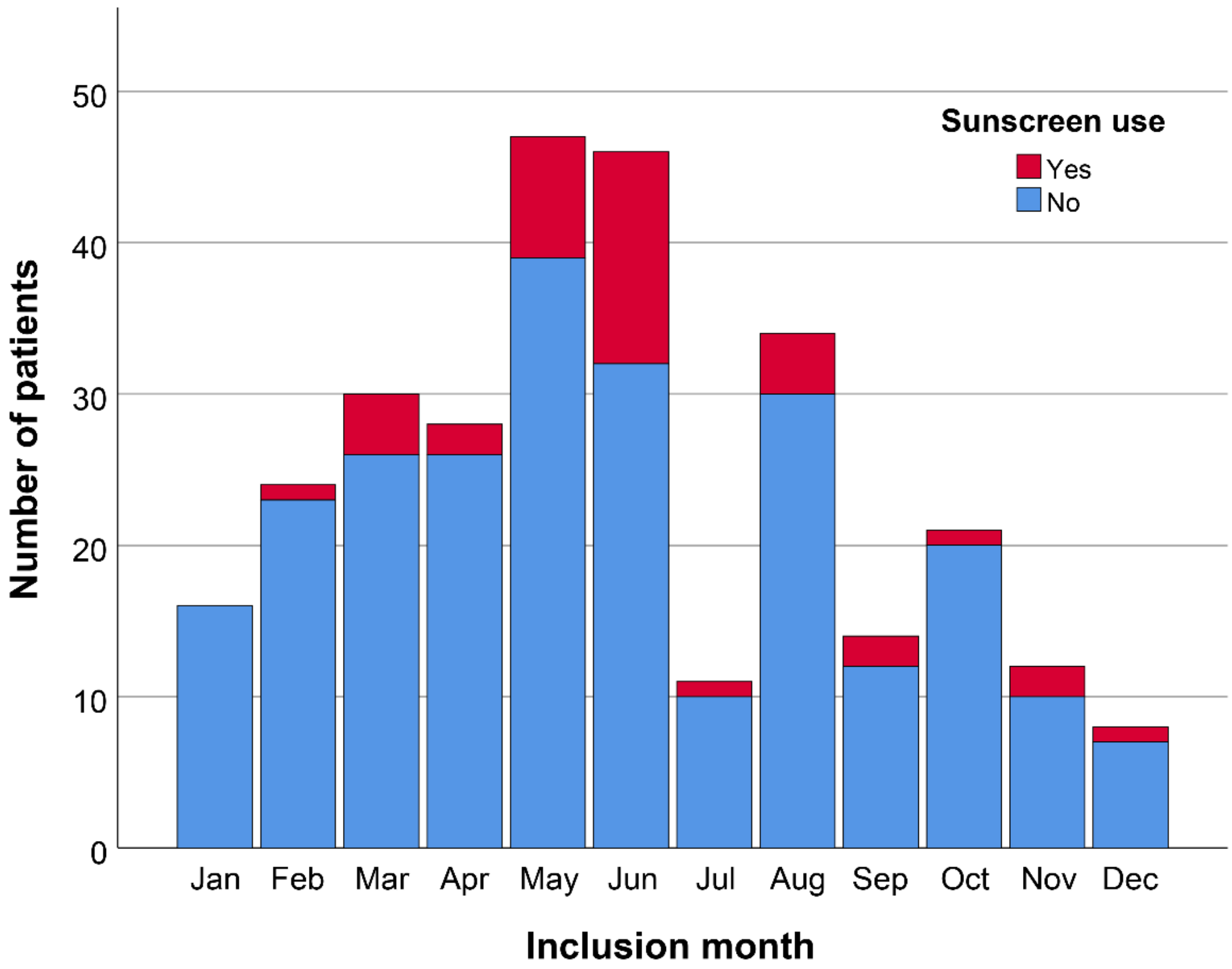

Figure S1. Number of SOTRs included in the study by month of the year. SOTRs excluded because of sunscreen use (red boxes) were predominantly enrolled during late spring and summer months. Only brown-spot score was depending on the month of inclusion in the study (*p*=0.023) with a higher score during summer months.

Wiegell Video S1.mp4

Video S1. VISIA Imaging System used to capture standardized, high-resolution facial images under controlled lighting with UV-light, cross-polarized light and standard flash. Images were taken frontally and at 33° angles from the left and right side. The images were then analyzed using the intrinsic VISIA software for wrinkles, texture, UV-spots, brown-spots, visible-spots and red-features.

Wiegell Video S2.mp4

Video S2. Pigmentation and Individual Typology Angel (ITA) objectively assessed using a skin colorimeter (DSM IV, Cortex Technology, Hadsund, Denmark). The skin colorimeter measures the intensity of light reflected from chromophores in the skin such as hemoglobin and melanin which allows quantification of erythema and pigmentation. It uses the CIE L* a* b* color space system and calculates the ITA, an objective classification of skin color.
